# Prevalence of Bloodstream Pathogens Is Higher in Neonatal Encephalopathy Cases vs. Controls Using a Novel Panel of Real-Time PCR Assays

**DOI:** 10.1371/journal.pone.0097259

**Published:** 2014-05-16

**Authors:** Cally J. Tann, Peter Nkurunziza, Margaret Nakakeeto, James Oweka, Jennifer J. Kurinczuk, Jackson Were, Natasha Nyombi, Peter Hughes, Barbara A. Willey, Alison M. Elliott, Nicola J. Robertson, Nigel Klein, Kathryn A. Harris

**Affiliations:** 1 Clinical Research Department, Faculty of Infectious & Tropical Diseases, London School of Hygiene & Tropical Medicine, London, United Kingdom; 2 MRC/UVRI Uganda Research Unit on AIDS, Entebbe, Uganda; 3 Institute for Women’s Health, University College London, Medical School Building, London, United Kingdom; 4 Institute for Child Health, University College London, London, United Kingdom; 5 Mulago University Hospital, Kampala, Uganda; 6 National Perinatal Epidemiology Unit, Nuffield Department of Population Health, University of Oxford, Oxford, United Kingdom; 7 Department of Microbiology, Virology and Infection Control, Great Ormond Street Hospital for Children, London, United Kingdom; Wadsworth Center, United States of America

## Abstract

**Background:**

In neonatal encephalopathy (NE), infectious co-morbidity is difficult to diagnose accurately, but may increase the vulnerability of the developing brain to hypoxia-ischemia. We developed a novel panel of species-specific real-time PCR assays to identify bloodstream pathogens amongst newborns with and without NE in Uganda.

**Methodology:**

Multiplex real-time PCR assays for important neonatal bloodstream pathogens (gram positive and gram negative bacteria, cytomegalovirus (CMV), herpes simplex virus(HSV) and *P*. falciparum) were performed on whole blood taken from 202 encephalopathic and 101 control infants. Automated blood culture (BACTEC) was performed for all cases and unwell controls.

**Principal Findings:**

Prevalence of pathogenic bacterial species amongst infants with NE was 3.6%, 6.9% and 8.9%, with culture, PCR and both tests in combination, respectively. More encephalopathic infants than controls had pathogenic bacterial species detected (8.9%vs2.0%, p = 0.028) using culture and PCR in combination. PCR detected bacteremia in 11 culture negative encephalopathic infants (3 Group B Streptococcus, 1 Group A Streptococcus, 1 *Staphylococcus aureus* and 6 Enterobacteriacae). Coagulase negative staphylococcus, frequently detected by PCR amongst case and control infants, was considered a contaminant. Prevalence of CMV, HSV and malaria amongst cases was low (1.5%, 0.5% and 0.5%, respectively).

**Conclusion/Significance:**

This real-time PCR panel detected more bacteremia than culture alone and provides a novel tool for detection of neonatal bloodstream pathogens that may be applied across a range of clinical situations and settings. Significantly more encephalopathic infants than controls had pathogenic bacterial species detected suggesting that infection may be an important risk factor for NE in this setting.

## Introduction

Identification of bloodstream infections in newborns, either alone or as a co-morbidity amongst infants affected by conditions such as neonatal encephalopathy (NE), is problematic. Clinical signs associated with neonatal sepsis syndrome, such as lethargy, poor feeding and convulsions, overlap substantially with other conditions such as NE. In preclinical studies exposure to bacterial endotoxin has been shown to increase vulnerability of the developing brain to perinatal hypoxia-ischemia [1]–[3]. However, clinical studies have yet to precisely define the relationship between perinatal infections and neonatal encephalopathy (NE). Fetal exposure to inflammation and, or, infection has also been found to increase brain vulnerability to hypoxia-ischemia via stimulation of toll-like receptors, immune and inflammatory responses, chemotaxis and cell death[4]–[6]. If implicated, the high prevalence of perinatal infections in the African setting means the contribution to the etiology of NE may be substantial.

The use of pediatric blood culture techniques in the neonatal period is complicated by low circulating bacterial load, the small volumes of blood taken, and because intrapartum antibiotics may render bacteria non-viable. Open techniques for taking neonatal blood samples increase the risk of contamination with commensal organisms making interpretation of results problematic. In contrast, real-time PCR techniques need less blood (<500 ul) and can detect the presence of non-viable bacteria. Additionally, real-time PCR assays are semi-quantitative, facilitating differentiation of clinically significant results from contamination.

Despite being a potentially useful adjunct to culture, real-time PCR for the diagnosis of typical sepsis pathogens from blood in any group is not frequently reported in the literature. The majority of studies using molecular techniques to diagnose bloodstream infections in neonates have focused on broad-range bacterial PCR assays, mainly those targeting 16 S rDNA [7]. However, despite the ability of such assays to detect a wide range of bacterial species their sensitivity is limited by ubiquitous contamination of PCR reagents with bacterial DNA [8], [9]. Well-designed and optimized species-specific real-time PCR assays offer 100-fold greater sensitivity [10].

Our aim was to develop a novel panel of species-specific real-time PCR assays to identify bloodstream pathogens in term infants with NE in Uganda and to compare the prevalence of pathogenic bacterial species between encephalopathic infants and unaffected controls.

## Methods

### Ethics Statement

London School of Hygiene & Tropical Medicine, Uganda Virus Research Institute (UVRI) and the Uganda National Council on Science and Technology ethics committees approved the study. Informed written parental consent was taken for all study participants.

Infants were recruited to a larger epidemiological study examining perinatal risk factors for NE in Uganda (the ‘ABAaNA study’) September 2011 through October 2012. Infants were recruited at Mulago Hospital, Kampala, Uganda’s National Referral Hospital. Participants were in-born term infants, <12 hours of age. Cases were infants with NE defined according to the Thompson clinical scoring system [11] with case infants scoring ≥6 and controls ≤3. Concurrently recruited unmatched controls were randomly selected from labor ward admissions. Exclusion criteria included living >20 km radius of Mulago Hospital, neonatal intravenous antibiotics administered prior to recruitment and parental consent declined.

Aseptic non-touch venepuncture was performed by trained clinical study staff: 0.5 ml blood (EDTA microfuge tube) for quantitative PCR (all infants), 0.5–1.0 ml whole blood inoculated into a single pediatric blood culture (BC) bottle (BACTEC Peds Plus, Becton Dickinson, Sparks MD; all cases and control infants clinically unwell on assessment), and 0.5 ml (serum microfuge tube) for C-reactive protein (CRP) (all infants, COBAS, Roche Diagnostics). A repeat sample for CRP was taken after 48 hours amongst surviving cases. Culture bottles were loaded into the Bactec 9240 automated instrument at the MRC/UVRI microbiology laboratory. Gram-stained, subcultured, and purified colonies were manually identified from positive cultures.

Bead-beating of EDTA-blood samples (300 µl) was performed to ensure complete lysis of bacterial cells. DNA (200 µl elute) was extracted using the QiAmp DNA blood mini kit and a QIAcube semi-automated extraction platform (both Qiagen, Manchester, UK). Each batch (12 samples) included a negative extraction control (200 µl of buffer AE, Qiagen). CT and PN performed all DNA extraction in the MRC/UVRI virology laboratories.

Ten targets and an internal control were amplified in five separate multiplexed reactions (Table 1) on a 7500 Fast Real Time PCR system (Life Technologies, Paisley, UK). Cycling conditions were 95°C for 5 minutes then 45 cycles of 95°C for 30 seconds and 60°C for 30 seconds. A positive result was any target detected with a cycle-threshold (CT) value <38. A no template control was included for each multiplexed assay. A positive control was included for every target organism. For bacterial targets this was DNA extracted from suspensions of cultured laboratory strains of known provenance, for viral targets this was plasmid DNA and for *Plasmodium falciparum* this was a microscopically positive clinical sample. A valid run required detection of each positive control with a CT value within a set range (normally 28.5–31.5). For bacterial assays suspensions of control organisms were quantified using a plate-counting method and for viral assays plasmid DNA was quantified using the NanoDrop Spectrophotometer (Labtech, Uckfield, UK). PCR assays were performed in the laboratories of UVRI and Great Ormond Street Hospital (GOSH), by CT and KH. Clinical staff and technicians were not blinded to case control status. Blood culture results were not available to staff prior to performing PCR assays.

**Table 1:** Primer and probe target genes, sensitivities and sequences for the Real-Time PCR assays.

Organism	Target gene	Assay sensitivity	Reference	Primer and probe sequences	Multiplex Reaction Mixtures
*Staphylococcus aureus*	coa	0.1–1 cfu/reaction	Sabet 2006	coa-forward: 5′-GTAGATTGGGCAATTACATTTTGGAGG	**Mix 1**: 1X QuantiFast Multiplex PCR mastermix (Qiagen), 0.15 µM each of coa-forward, coa-reverse and coa-probe, 0.3 µM each of GAS-forward and GAS-reverse, 0.2 µM GAS-probe, 0.1 µM each of IPC-forward, IPC-reverse, IPC-probe, 7 µl extracted DNA and molecular grade water to give a final reaction volume of 20 µl.
				coa-reverse: 5′-CGCATCTGCTTTGTTATCCCATGTA	
				coa-probe: 5′FAM- TAGGCGCATTAGCAGTTGCATC-BHQ1	
*Streptococcus pyogenes*	*csrR*	0.1–1 cfu/reaction	This study	GAS-forward: 5′-TGGATGTGGTTGCAGGTTTAGAC	
				GAS-reverse: 5′- CGGGCAAGTAGTTCTTCAATGG	
				GAS-probe: 5′-JOE- CGGTGCAGACGACTATATTGTTAAACC-BHQ1	
InternalPositivecontrol (IPC)	*Mus* non-coding sequencing	NA	This study	IPC-forward: 5′-GGACACTATGCCCCTCCTTAGA	
				IPC-reverse: 5′-AGCTCCAAACTCCGTCTCTGTAA	
				IPC-probe: 5′Cy5-TTGGGAACAAAACACCCATGGAAGGA-BHQ2	
*Streptococcus pneumoniae*	*lytA*	0.1–1 cfu/reaction	Harris et al 2008	lytA-forward: 5′-ACGCAATCTAGCAGATGAAGC	**Mix 2**: 1X QuantiFast Multiplex PCR mastermix (Qiagen), 0.2 µM each of lytA-forward, lytA-reverse, lytA-probe, GBS-forward, GBS-reverse and GBS-probe, 7 µl extracted DNA and molecular grade water to give a final reaction volume of 20 µl.
				lytA-reverse: 5′- TGTTTGGTTGGTTATTCGTGC	
				lytA-probe: 5′FAM- TTTGCCGAAAACGCTTGATACAGGG-BHQ1	
*Streptococcus agalactiae*	*sip*	0.1–1 cfu/reaction	Berseng et al 2007/Probe this study	GBS-forward: 5′-ATCCTGAGACAACACTGACA	
				GBS-reverse: 5′- TTGCTGGTGTTTCTATTTTCA	
				GBS-probe: 5′ JOE- ATCAGAAGAGTCATACTGCYACTTC-BHQ1	
Enterobacteriaceae family	*dnaK*	1–10 cfu/reaction	This study	ent-forward: 5′-ACCTGGGTACWACCAACTCTTGTGT	**Mix 3**: 1X QuantiFast Multiplex PCR mastermix (Qiagen) 0.25 µM each of ent-forward, ent-reverse and ent-probe, 0.1 µM each of tuf-forward, tuf-reverse and tuf-probe, 7 µl extracted DNA and molecular grade water to give a final reaction volume of 20 µl.
				ent-reverse: 5′-GTCACTGCCTGACGTTTAGC	
				ent-probe: 5′-JOE-AGGATGGTGAAACTCTGGTWGGTCAGCC-BHQ1	
*Staphylococcus species*	*tuf*	1–10 cfu/reaction	This study	tuf-forward: 5′-CATTCCAACTCCAGAACGTGAYT	
				tuf-reverse: 5′-CACGACCAGTGATTGAGAATACG	
				tuf-probe: 5′-CY5-TGAYAAACCATTCATGATGCCAGTTGAGG-BBQ	
CMV	DNA polymerase	5 copies/reaction	This study	CMV-forward: 5′-GCATGCGCGAGTGTCAAGAC	**Mix 4**: 1X QuantiFast Multiplex PCR mastermix (Qiagen), 0.2 µM each of CMV-forward, CMV-reverse, CMV-probe, P. fal-forward, P. fal-reverse and P. fal-probe, 7 µl extracted DNA and molecular grade water to give a final reaction volume of 20 µl
				CMV-reverse: 5′-GTTACTTTGAGCGCCATCTGTTCCT	
				CMV-probe: 5′-CY5-TGCGCCGTATGCTGCTCGACA-BBQ	
Plasmodium falciparum	SSU RNA	<3parasites/ul	Adegnika et al 2006	PFal-forward: 5′-CCG ACT AGG TGT TGG ATG AAA GTG TTA A	
				Plas-reverse: 3′-AAC CCA AAG ACT TTG ATT TCT CAT AA	
				Pfal-probe: 5′- FAM-CTT TCG AGG TGA CTT TTA GAT-	
HSV 1 and 2	DNA polymerase	5 copies/reaction	This study	HSV-forward: 5′-GACAGCGAATTCGAGATGCTG	**Mix 5**:1XQuantiFast Multiplex PCR mastermix (Qiagen), 0.4 µM each of HSV-forward and HSV-reverse, 0.2 µM each of HSV-1-probe and HSV-2-probe, 7 µl extracted DNA and molecular grade water to give final reaction volume of 20 µl.
				HSV-reverse: 5′-ATGTTGTACCCGGTCACGAACT	
				HSV1-probe: 5′-FAM-CATGACCCTTGTGAAACA-MGB	
				HSV-2-probe: 5′-VIC-TGACCTTCGTCAAGCAG-MGB	

Enterobacteriaceae PCR positives were further identified by amplicon sequencing using Big-Dye 3.1 Cycle-sequencing kit (Life Technologies) and analyzed on a 3130 Genetic Analyse (Life Technologies). Sequences were compared to an in-house database of Enterobacteriaceae *dnaK* sequences using MegAlign (Lasergene 10, DNAStar, Madison, WI, USA) or by BLAST searching against the Genbank database (http://www.ncbi.nlm.nih.gov).

The prevalence of bacteremia between cases and controls was compared using the Chi-squared test using STATA version 12.0.

## Results

**Figure 1:**
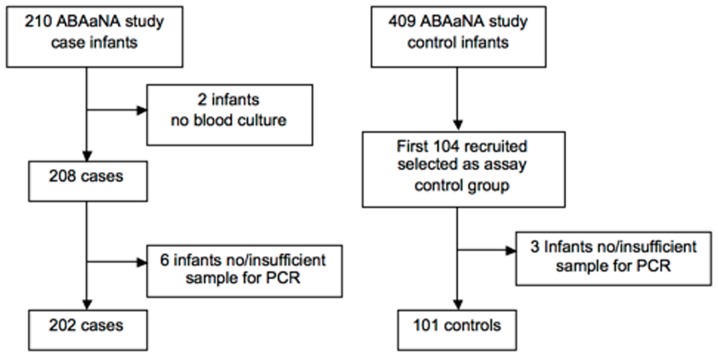
Flow diagram of study case and control participant.

Samples from 202 case and 101 controls were examined (Figure 1). Most encephalopathic infants were moderately or severely affected (89.1%, n = 180). Compared to controls they were more likely to be male, have abnormal APGAR scores at one and five minutes, and have a higher mean birth weight (Table 2). Intrapartum antibiotic use was similar.

**Table 2:** Baseline demographic and clinical characteristics of mothers and babies.

Characteristic	Case	Control	P-value^a^
	N(%)	N(%)	
**Maternal**			
Maternal education≤primary level	79/202 (39.1)	45/100 (45.0)	0.327
Nulliparity	118/202 (58.4)	49/101 (48.5)	0.102
Emergency caesarean section	49/201(24.4)	23/101(22.8)	0.757
Intrapartum antibiotics	37/201 (18.4)	16/101 (15.8)	0.580
**Neonatal**			
Male sex	132/202 (65.4)	48/101 (47.5)	0.003
Mean birth weight (g)	3183	3033	0.0061
1 minute APGAR <3	65/195 (33.3)	1/99 (1)	<0.0001
5 minute APGAR <7	126/177 (71.2)	1/98 (1)	<0.0001
Case fatality	68/200(34.0)	0/96 (0.0)	<0.0001

a chi-squared test.

### Comparison of Bacterial PCR and Culture Results amongst Infants with NE vs. Controls

The prevalence of neonatal bacteremia with a pathogenic organism amongst encephalopathic infants was 3.5% (7) by BC alone, 6.9% (14) by PCR alone and 8.9% (18) by BC and PCR in combination (Table 3). Of the 101 control infants, none was positive for pathogenic bacterial species on BC but two were positive on PCR, giving a prevalence of neonatal bacteremia amongst the control group of 2.0% (2/101). Four control infants were clinically unwell on recruitment, so BCs were performed in addition to PCR; all 4 were negative on both tests. The prevalence of bacteremia (by either BC or PCR) was significantly higher amongst infants with encephalopathy than those without (8.9% vs. 2.0% respectively, p-value = 0.028). Coagulase negative staphylococcus (CoNS), usually considered non-pathogenic amongst term newborns, was more commonly found on PCR amongst controls compared to cases, but this difference was not statistically significant (9.9% (9/101) in controls vs. 5.5% (11/202) in cases, p = 0.252).

**Table 3:** Comparison of species specific bacterial qPCR and BACTEC blood culture for detection of pathogenic bacteria in blood samples amongst neonatal encephalopathy case infants.

	Blood Culture		
	Positive	Negative	
**PCR Positive**	3	11	14
**PCR Negative**	4	184	188
	7	195	202

### Case Infants with Positive Blood-cultures

Seven case infants had a positive BC, four of these (3 *Staphylococcus aureus* and 1 Group C Streptococcus) were PCR negative. The other three cases also yielded a positive PCR result; one *E. coli* by both methods, a second *S. aureus* by both methods but also PCR positive for the Enterobacteriaceae target and a third case was BC positive for CoNS but PCR positive for Group B Streptococcus (GBS) (Table 4).

**Table 4:** Results of blood culture, real-time PCR assays and sequencing of positives.

Patient	Blood culture	PCR	Sequence	Intrapartum antibiotics	CRP	CRP
					Day 1	Day 3
**GROUP B STREPTOCOCCUS**						
Case 61	Coagulase Negative Staphylococcus	Group B Streptococcus	–	Yes	16.86	50.4
Case 81	Negative	Group B Streptococcus	–	No	47.52	Died D1
Case 170	Negative	Group B Streptococcus	–	No	4.23	Died D1
**OTHER STREPTOCOCCUS**						
Case 133	Negative	Group A Streptococcus	–	No	61.16	Died D1
Case 150	Group C Streptococcus	Negative	–	No	0.28	1.02
**STAPHYLOCOCCUS AUREUS**						
Case 37	*S. aureus*	Negative	–	No	6.22	4.03
Case 83	Negative	*S. aureus*	–	No	2.28	30.4
Case 157	*S. aureus*	Negative	–	Yes	81.55	63.06
Case 166	*S. aureus*	Negative	–	No	8.18	20.41
Control 5	Not performed	*S. aureus*	–	No	55.56	n/a
**GRAM NEGATIVE ORGANISMS**						
Case 41	Negative	Enterobacteriaceae	*Enterobacter* sp.	No	7.58	insuff
Case 53	*E.Coli*	Enterobacteriaceae	*E.coli* or *Klebsiella* sp.	No	1.1	Died D1
Case 56	Negative	Enterobacteriaceae	Failed	No	0.47	Died D1
Case 64	Negative	Enterobacteriaceae	Failed	No	0.22	13.16
Case 67	Negative	Enterobacteriaceae	*Enterobacter* sp.	No	0.14	Died D1
Case 82	Negative	Enterobacteriaceae	*Pantoea* sp.	No	8.91	Died D1
Case 117	Negative	Enterobacteriaceae	*E.Coli*	Yes	32.81	Died D1
**MULTIPLE ORGANISMS**						
Case 132	*S. aureus*	*S. aureus*	–	No	49.01	–
		Enterobacteriaceae	*E.Coli*			
		Cytomegalovirus	–			
Case 49	*Klebsiella* sp.	Enterobacteriaceae	*Klebsiella sp*.	Yes	3.61	9.32
		*P. falciparum*	–			
Control 65	Not performed	Enterobacteriaceae	*E.Coli*	No	1.83	n/a
		*S. pneumoniae*	–			
**VIRUSES & MALARIA**						
Case 10	Negative	Cytomegalovirus	–	n/a	2.68	Died D1
Case 16	Negative	Cytomegalovirus	–	n/a	0.16	Died D1
Control 21	Not perform	Cytomegalovirus	–	n/a	0.32	n/a
Case 175	Negative	Herpes Simplex Virus 1	–	n/a	0.35	2.79
Control 98	Not performed	*P. falciparum*	–	n/a	1.38	n/a

### Cases with Negative Blood-cultures

One hundred and ninety five case infants had negative BCs, 184 of these were also negative by PCR.

The remaining 11 BC negative cases were positive by real-time PCR for a bacterial target (3 GBS, 1 Group A Streptococcus (GAS), 1 *S. aureus* and 6 Enterobacteriaceae). Of the five infants PCR positive, BC negative for pathogenic streptococcal or staphylococcal species, four had substantially raised CRP levels on day 1 or day 3 and the fifth (case 170) died <24 hours after a clinical course consistent with aggressive GBS septicemia with persistent hyperthermia, severe respiratory distress and seizures. Sequencing of the Enterobacteriaceae amplicon in the 6 positive cases allowed identification of a pathogenic organism (at least to genus level) in 4 cases (2 *Enterobacter sp*., 1 *Pantoea sp*. and 1 *E. coli*) but was unsuccessful in 2 cases therefore these are identified only as Enterobacteriaceae (Table 4).

In the absence of normative CRP data for this population, a raised CRP was defined as CRP>97^th^centile amongst controls ( = 40.7 mg/L). Of case infants without evidence of bacteremia on PCR or BC, 5.0% (9/181) had a raised CRP on day one, rising to 16.2% (19/117) on day 3 amongst survivors. Bacteraemic case infants were significantly more likely to have a raised CRP on day one compared to non-bacteraemic infants (22.2% (4/18) vs. 5.0% (9/181), p = 0.005).

### Cytomegalovirus, Herpes Simplex & Malaria

The prevalence of cytomegalovirus (CMV) infection was 1.5% (3/202) amongst cases and 1.0% (1/101) in controls. Herpes simplex virus (HSV) was identified in one case infant (0.5%) and *P. falciparum* in one case and one control infant.

## Discussion

The accurate diagnosis of infectious co-morbidity in the unwell newborn through clinical signs or standard culture techniques is problematic [12]. Our novel panel of multiplexed real-time PCR assays, designed to cover the majority of common pathogens implicated in congenital neonatal infections, was successful in detecting more bacteremia than culture alone amongst encephalopathic Ugandan newborns. A significantly higher prevalence of potentially pathogenic bacterial species was seen in encephalopathic babies when compared to controls suggesting that fetal exposure to bacterial blood-borne pathogens may be a causal factor for neonatal encephalopathy in this setting. This may be mediated by cytokines and inflammatory cells which have been found to be intermediaries in perinatal brain injury secondary to hypoxia-ischemia, neurotoxins and infection [13] or by enhanced sensitivity of the brain to hypoxia-ischemia after exposure to bacterial products such as endotoxin^3^.

Eleven culture-negative encephalopathic infants tested positive on bacterial PCR for pathogenic organisms. Amongst these, two organisms could only be identified and as Enterobacteriaceae and as this family contains environmental non-pathogenic species, interpretation of these two PCR positives is uncertain. Coagulase negative staphylococcus, considered non-pathogenic in the absence of indwelling catheters, was common amongst both case and control infants. Open venepuncture may have contributed to an increased contamination risk. False positive PCR results due to laboratory contamination are unlikely since negative controls were consistently negative and strict laboratory practice was followed to prevent contamination.

The prevalence of congenital cytomegalovirus (CMV) is thought to be high in low income settings (1–5%) where it is commonly secondary to non-primary maternal infections [14]. No Ugandan studies have published rates of congenital CMV infection. Our prevalence of 1% amongst controls is substantially lower than that reported from a recent birth cohort in the Gambia (5.4%) [15]. Our prevalence of congenital CMV was similar between encephalopathic and non-encephalopathic infants suggesting that CMV does not contribute to the burden of neonatal encephalopathy in this setting. Herpes Simplex and *P. falciparum* infections were similarly uncommon.

There are limitations of all laboratory techniques utilized in the detection of bacteremia. In our study, important pathogens such as GBS, *S. aureus* and Enterobacteriaceae were detected by PCR but not culture; however bacteremia in four infants was detected by culture alone. This was most common for *S. aureus*, a robust organism that grows well in the laboratory hence culture may be more sensitive than PCR for detecting low levels of *S. aureus* bacteremia in these infants. Even a combination of PCR and culture is unlikely to exclude all infants with early sepsis, highlighted by the substantial number of culture negative/PCR negative infants with a substantially raised CRP on either day one or day 3. This emphasizes the importance of empirical antibiotic therapy to all sick newborns including those with NE. In addition, PCR assays offer no information on antimicrobial susceptibility, and contamination with commensal and environmental organisms remains an issue for all techniques.

In conclusion, we have found high levels of bacteremia in infants with NE in an African setting using a combination of blood culture and species-specific real-time PCR assays to identify bloodstream pathogens. Significantly more encephalopathic infants than controls had pathogenic bacterial species detected suggesting that neonatal bacteremia may be an important risk factor for NE in this setting. Our real-time PCR panel detected more bacteremia than culture alone and provides a novel tool for detection of neonatal bloodstream pathogens that could be applied across a range of clinical situations and settings.
